# Biowaste and by-products as rearing substrates for black soldier fly (*Hermetia illucens*) larvae: Effects on larval body composition and performance

**DOI:** 10.1371/journal.pone.0275213

**Published:** 2022-09-29

**Authors:** Kylian Manon Eggink, Ivar Lund, Per Bovbjerg Pedersen, Benni Winding Hansen, Johanne Dalsgaard

**Affiliations:** 1 Section for Aquaculture, The North Sea Research Centre, Technical University of Denmark, Hirtshals, Denmark; 2 Department of Science and Environment, Roskilde University, Roskilde, Denmark; Universita degli Studi della Basilicata, ITALY

## Abstract

Black soldier fly (*Hermetia illucens*) larvae can convert biowaste and by-products into body mass high in protein (~40% dry matter, DM) and lipid (~30% DM). However, the type of rearing substrate also affects the larval body composition and thus its nutritional value. Hitherto, it remains unclear how and to what extent the larval body composition can be altered by the substrate. This study was therefore performed to examine the possibilities of modifying larval body composition using different rearing substrates. To investigate this, 5-days old larvae were reared for seven days on different locally available waste and by-products: brewer’s spent grain, mitigation mussels (*Mytilus edulis*), rapeseed cake, and shrimp waste meal (*Pandalus borealis*). Larval composition and performance were compared to larvae reared on a commercial chicken feed as well as a mixed feed (mixture of chicken feed and by-products, with a similar macronutrient composition to chicken feed). Larval body weight was recorded daily to determine growth over time whereas larvae and substrates were sampled at the start and end of the trial and analysed for their nutritional composition. The type of rearing substrate affected both larval body composition and growth performance. There was a clear relation between the nutritional composition of the substrate and larvae for certain fatty acids. Larvae reared on marine-based waste substrates contained a higher share of omega-3 fatty acids than larvae reared on the other substrates, indicating an accumulation of omega-3 fatty acids from the substrate. There was a strong positive linear correlation between the ash content in the substrate and larvae whereas larval lipid, protein, amino acid, and chitin content seemed more affected by larval development. Overall, this study showed that the rearing substrate affects larval composition and development, and that larval composition of certain nutrients can be tailored depending on further food and feed applications.

## 1. Introduction

One of the biggest challenges of our time is sustainable and sufficient food production, both in terms of quality and quantity, to meet growing demands. The increasing demands are driven by world population growth as well as dietary alterations due to socio-economic factors including urbanisation and economic growth [1]. However, these increased demands will put additional pressure on valuable resources (water, energy, etc.) whilst simultaneously leading to an increase in environmental impact [2]. Concurrently, global food waste is a major challenge due to both its large volumes and fast decomposition. Worldwide food waste volumes are circa 1.3 billion tons per year, which corresponds to approximately one-third of the food produced worldwide, and these estimates do not even include unavoidable biowaste from parts of food products that are inedible to humans (e.g. peels, bones, and shells) [3]. One way to deal with food waste and other biowaste, whilst obtaining high-quality nutrients, is through bioconversion using insects to convert waste into insect biomass.

Insects are a valuable nutrient source that can be used directly for human consumption or indirectly as feed for livestock [4]. One insect species that has been recognised as particularly promising is the black soldier fly (BSF, *Hermetia illucens*). This is due to its short life cycle, ability to handle a wide range of challenging environments (e.g. high temperatures, low food availability, and low oxygen) and capacity to consume a great variety of substrates during its larval stage [5]. Additionally, BSF larvae can ingest large quantities of substrates and convert them into body tissue with a high crude protein (38.3–52.3% dry matter, DM) and crude lipid (21.8–38.6% DM) content [6–9]. Due to the nutritional content of BSF larvae, they have been successfully incorporated into food [10, 11] and feed [12, 13], consolidating their value as an alternative nutrient source. However, the nutritional composition of BSF larvae can largely vary, depending on the different abiotic and biotic factors during rearing, with the substrate being among the most critical factors [8, 14].

Numerous studies have researched the use of biowaste and by-products for rearing BSF larvae such as animal manure, brewery by-products, fish offal, and fruit and vegetable waste [e.g. 8, 14–17]. These studies have shown that the larval composition and performance are largely modified by the rearing substrate. However, there is so far a limited and contradictory understanding of the relation between substrate and larvae nutrient composition. For example, some studies found that substrates high in protein resulted in larvae high in protein [14, 18] whereas another study found no such relation [8, 19, 20]. Additionally, it is currently unknown whether the larval chitin content can be modified via the rearing substrate, which could be important for future food and feed applications, as chitin and its derivatives have, amongst others, antimicrobial and immunomodulatory [21–23] as well as anti-nutritional [24, 25] properties.

The current study therefore aimed at identifying whether—and to what extent—the BSF larval nutritional composition of chitin and other nutritional components (DM, ash, protein, amino acids, lipid, and fatty acids) can be manipulated by individual rearing substrates using locally available biowaste and by-products for food and feed applications.

## 2. Materials and methods

### 2.1. Experimental animals

The study was carried out at a commercial insect company (ENORM Biofactory, Flemming, Denmark) using larvae from a BSF colony originally established in 2018. After hatching, BSF larvae were raised for 5 days by ENORM on a mixed feed with a similar macronutrient composition as commercial chicken starter feed but including by-products, consisting of 66.5% water, 16.0% pea grits, 8.0% wheat, 7.0% chicken starter feed, 2.1% sugar beet pellet, and 0.4% vitamin-mineral mixture. During these initial 5 days, the larvae were reared in a climate room maintained at constant conditions (34 °C and 70% relative humidity). Afterwards, larvae were sieved from the substrate and their average weight was determined by weighing 10 batches each of 500 hand-counted larvae. Thereafter, 18 subsamples were taken of approximately 10 000 larvae for the 7-day substrate trial.

### 2.2. Experimental substrates

Six different rearing substrates were tested during the substrate trial being the (1) same mixed feed used for the first 5 days of larval rearing, (2) commercial chicken starter feed with wheat and soybean meal as main ingredients (Hornsyld Købmandsgaard A/S, Hornsyld, Denmark), (3) rapeseed cake obtained as a by-product from rapeseed oil production (Emmelev A/S, Otterup, Denmark), (4) brewer’s spent grain obtained as a by-product from the brewing industry (Carlsberg A/S, Fredericia, Denmark), (5) mitigation mussels (*Mytilus edulis*) including tissues and shells that were grown to reduce eutrophication (Danish Shellfish Centre, Nykobing Mors, Denmark), and (6) shrimp waste (*Pandalus borealis*) derived from shrimps processed for human consumption including heads, appendages, and exoskeletons (Launis A/S, Skagen, Denmark).

Mitigation mussels and shrimp waste were individually mixed using a laboratory homogeniser (1094, Perstorp Analytical, Höganäs, Sweden) and ground to <5 mm using a meat mincer (TS12E, OMAS, Oggiona S. Stefano, Italy). Substrates were analysed for moisture content using a VWR moisture analyser and substrate moisture content of rapeseed cake and chicken feed was adjusted to ~70% using tap water whereas for the other substrates the moisture content was already within the optimal range (60–80%). Furthermore, 2% sugar beet pellets were added to each substrate to prevent the accumulation of freestanding water that risks drowning the larvae. Substrates were mixed thoroughly by hand and stored at -20 °C until further analysis. Before the trial, the substrates were thawed at room temperature for 24 hours.

### 2.3. Experimental design and data collection

The substrate trial was carried out as a randomised single-factor experiment with the six substrates fed to triplicate plastic boxes. Each box (59L x 39W x 31H cm) was covered with secured mesh fabric to prevent larvae from escaping. Boxes were stacked randomly and placed on plastic pallets (1 m above the floor) situated in an aerated room at 27.4 ± 0.8 °C and 67.7 ± 4.7% relative humidity, as previously recommended by Diener et al. [6]. At the start of the trial, 100 mg substrate larvae/day was added to each box with approximately 10 000 of 5-days old larvae placed on top of the substrate. The total substrate provided was corrected for subsequent daily samplings of substrate and larvae. Larvae were kept in complete darkness except when samples were taken.

Samples of larvae (n = 500 per box) were obtained daily using forceps. Individual larvae were washed with distilled water to remove substrate remnants and dried with tissue paper. Average body weights were subsequently determined by weighing the larvae in groups of 10 and sampled larvae were frozen at -20 °C until further analysis.

Daily, 10 g of substrate was taken for pH measurements using the protocol by Lalander et al. [26]. In brief, the sample was diluted with 50 mL distilled water, left for 1h at room temperature, and pH determined with a Lab 845 pH Meter (SI Analytics, Mainz, Germany). Simultaneously, substrate core temperature was measured using a Traceable Lollipop thermometer (VWR, Radnor, USA).

### 2.4. Analytical methods

Subsamples of substrates and larvae were freeze-dried (Christ Loc 2, Martin Christ Gefriertrocknungsanlagen GmbH, Osterode am Harz, Germany) and ground (A10 basic, IKA, Staufen, Germany) before determination of the content of DM, ash, protein, amino acids, lipid, fatty acids, and chitin (larvae only). DM content was obtained by drying samples at 105 °C until constant weight whilst ash content was determined by incineration at 550 °C until constant weight [27]. Crude protein was determined as Kjeldahl-N (ISO 2005) using a standard nitrogen-to-protein conversion factor (Kp) of 6.25. Additionally, corrected crude protein was determined for larvae using Kp values estimated by dividing the sum of amino acids by the total Kjeldahl-N as performed by Boulos et al. [28]. Crude lipid was quantified as described by Bligh and Dyer [29]. Gross energy was determined in larvae by combustion in a bomb calorimeter (C7000, IKA) while in substrates was estimated, due to insufficient sample quantity for calorimetric determination, assuming an energy content of 23.66 MJ/kg protein, 39.57 MJ/kg lipid, and 17.17 MJ/kg nitrogen-free extract (NFE) [30]. NFE (%_DM_) = 100 − (crude lipid %_DM_ + crude protein %_DM_ + ash %_DM_).

The amino acid contents of substrates and larvae and chitin content of larvae were quantified by HPLC and spectrophotometry, respectively, as previously described by Eggink et al. [31].

Prior to fatty acid determination, total lipids were extracted by incubating samples in chloroform-methanol (2:1) for 24 hours [32]. Subsequently, fatty acids were determined by gas chromatography (HP-5890A, Agilent Technologies, Santa Clara, USA) and separated on a column (Agilent DB wax 127–7012, 10m x 100μm x 0.1μm, Agilent Technologies). A standard mixture of fatty acid methyl esters (Nu Check Prep 68D, USA) was used for fatty acid identification. Fatty acids were reported as the area percentage of total identified fatty acids.

### 2.5. Statistical methods

Data are presented as mean ± standard error (SE), unless otherwise mentioned, considering each box an experimental unit. All data were tested for normality of residuals and homogeneity of variances using the Shapiro-Wilk and Levene’s tests, respectively. In instances where data were not normally distributed, arcsine (percentage data) or log (other data) transformation was performed. Larval proximate composition data were subjected to a one-way ANOVA to compare means between the different treatment groups. When differences between means were significant (p < 0.05), a Tukey honest significant difference *post hoc* test was performed. Data over time of larval growth, substrate composition, substrate temperature, and substrate pH over time were analysed using repeated measures ANOVA with ‘substrate’ as between-subject factor and ‘time’ as within-subject factor, to test for the effect of time and substrate. The relation between the nutrient content in substrate and larvae was analysed using the Pearson correlation coefficient. All statistical tests were performed using IBM SPSS Statistics 25.0 (IBM Corp., USA) while graphs were generated using GraphPad Prism 9.2.0 software (GraphPad Software, USA).

## 3. Results

### 3.1. Growth performance

**Fig 1:**
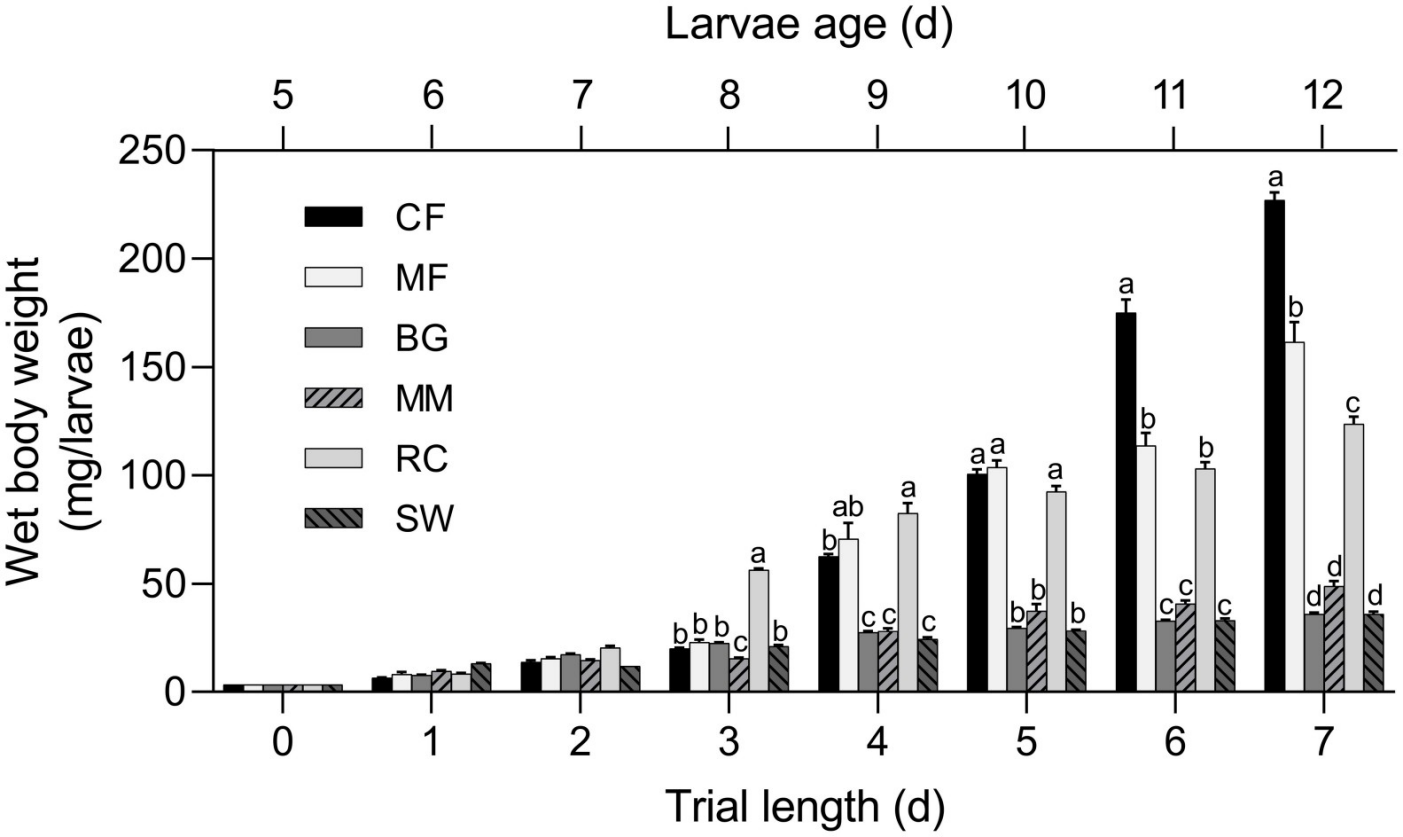
Mean ± standard error (n = 3) of individual wet body weight (mg) of black soldier fly larvae over time reared on six different rearing substrates: Chicken feed (CF), mixed feed (MF), brewer’s spent grain (BG), mitigation mussels (MM), rapeseed cake (RC), and shrimp waste (SW). Dissimilar lower case superscript letters represent significant differences (p < 0.05) between means within the same time point.

The effect of rearing substrate on BSF wet body weight over time is depicted in Fig 1. The average larval wet body weight was 3.5 ± 0.1 mg across all treatment groups at the start of the trial. On the third day of the trial, significant differences between treatments started to occur with larvae reared on rapeseed cake having a significantly higher body weight (56.5 ± 0.8 mg) whereas larvae reared on mitigation mussels having a significantly lower body weight (15.5 ± 0.6 mg) than the other treatment groups averaging from 20.1–23.0 mg. On day four, larval body weight was significantly higher for larvae fed chicken feed, mixed feed and rapeseed cake than for the rest of the treatment groups and this trend was maintained until the end of the trial. On the last day of the trial (day seven), larvae reared on chicken feed had the highest final body weight (227.1 ± 12.7 mg) followed by larvae reared on mixed feed (151.2 ± 18.2 mg) and rapeseed cake (123.9 ± 9.6 mg)—all three dietary treatment groups being significantly different from each other. A significantly lower final body weight was observed for larvae reared on mitigation mussels (48.9 ± 7.3 mg) followed by larvae reared on shrimp waste and brewers spent grain (36.0 ± 4.1 mg), although not significantly different from each other.

**Fig 2:**
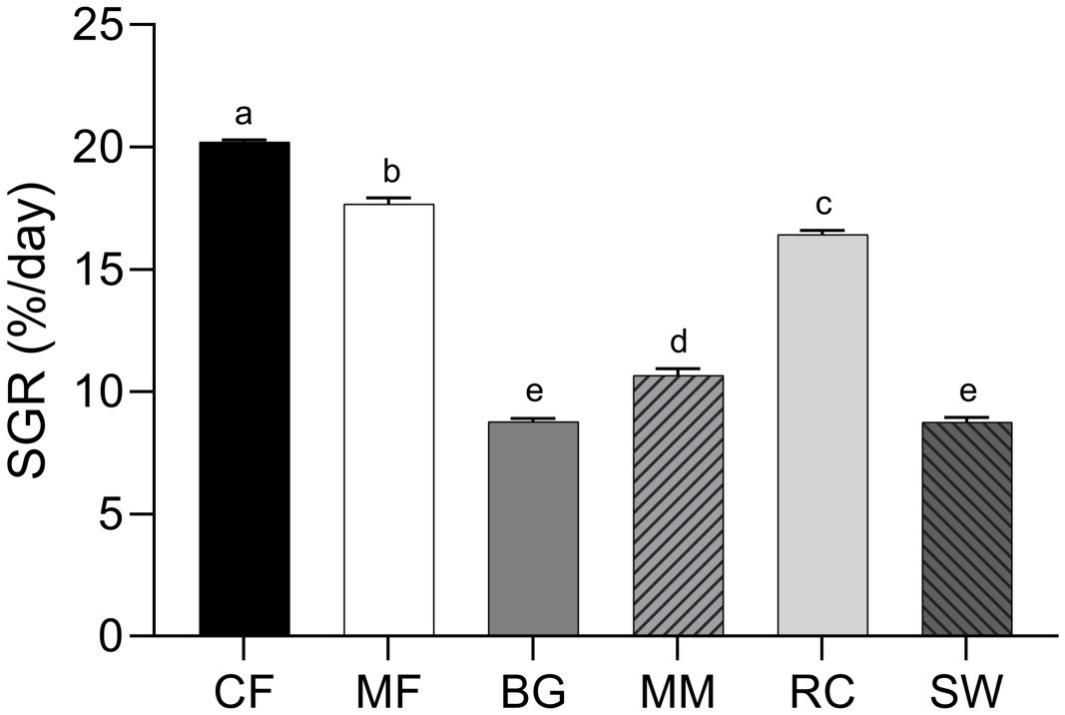
Mean ± standard error (n = 3) of specific growth rate (SGR, %/day) of black soldier fly larvae during the 7-day trial on six different rearing substrates: Chicken feed (CF), mixed feed (MF), brewer’s spent grain (BG), mitigation mussels (MM), rapeseed cake (RC), and shrimp waste (SW). Dissimilar lower case superscript letters represent significant differences (p < 0.05) between means. SGR (%/day) = (ln (final body weight (g))—ln (initial body weight (g)))/time of trial (d) * 100.

The specific growth rate (SGR) was used to evaluate the overall larval growth performance during the 7-day trial. The SGR was significantly higher for larvae fed chicken feed (20.2 ± 0.1%/d), followed by those fed mixed feed (17.7 ± 0.3%/d), rapeseed cake (16.4 ± 0.2%/d), and mitigation mussels (10.7 ± 0.3%/d)—all treatments being significantly different from each other (Fig 2). The lowest SGR was found for larvae fed brewer’s spent grain (8.8 ± 0.1%/d) and shrimp waste (8.8 ± 0.2%/d), values being significantly different from the other dietary treatment groups, but not from each other.

### 3.2. Larval composition

The initial and final body composition of the larvae are described in Table 1, while changes in larval protein and lipid composition over time can be found in S1 and S2 Figs, respectively. For all treatments, crude lipid significantly increased during the growth period and, aside from a few exceptions, this was also the case for energy and chitin. Whereas NFE significantly decreased during the same period, except for mixed feed. There were no clear over time changes for DM, ash, and crude protein. Additionally, the body composition of larvae harvested by the end of the trial was significantly affected by the rearing substrates. The crude protein content was significantly highest in larvae fed brewer’s spent grain (59.8% DM) and lowest in those fed mixed feed and chicken feed (43.4–43.6% DM). However, the crude protein content, calculated using the standard Kp factor of 6.25, was 21–32% higher than the corrected crude protein content calculated using Kp factors based on the total amino acid content determined in the larvae (**Table 4**). Larval crude lipid ranged from 21.6 to 32.9% DM and was significantly different between several of the treatment groups, with the highest lipid content found in larvae fed chicken feed and the lowest content in larvae fed mitigation mussels. Chitin in the larvae ranged from 2.6–5.1% DM and was lowest for larvae reared on mitigation mussels and highest for larvae fed the chicken feed and mixed feed. Larval energy content was lowest for 5-days old larvae and 12-days old larvae reared on mitigation mussels (16.7 and 17.7 kJ/g DM, respectively), and highest for those reared on chicken feed, rapeseed cake, and mixed feed (23.2–24.1 kJ/g DM). Larval ash content was also significantly affected by the substrate with the lowest content observed in larvae reared on the mixed feed (9.0% DM) and highest for those reared on mitigation mussels (22.9% DM). Nitrogen-free extract was highest in the initial larvae (23.0% DM) and lowest for final larvae reared on mitigation mussels (7.1% DM). There was a strong correlation between larval and substrate ash content (r = 0.97, p < 0.001, n = 18) while there were no similar correlations for protein (r = 0.37, p = 0.15, n = 18), lipid (r = -0.02, p = 0.93, n = 18), or DM (r = 0.37, p = 0.13, n = 18).

**Table 1:** Proximate composition of initial (5-days old) and final (12-days old) black soldier fly larvae reared on six different substrates (chicken feed (CF), mixed feed (MF), brewer’s spent grain (BG), mitigation mussels (MM), rapeseed cake (RC), and shrimp waste (SW)). All values are shown as mean ± standard error (SE; n = 3) for percentage of wet weight (%_WW_), percentage of dry matter (%_DM_), or kJ/g dry matter (DM). Corrected crude protein is calculated using the nitrogen-to-protein conversion factors described in **Table 4**. Dissimilar lower case superscript letters represent significant differences between means within the same column (p < 0.05).

Sample type	Dry matter (%_WW_)	Ash (%_DM_)	Chitin (%_DM_)	Crude protein (%_DM_)	Corrected crude protein (%_DM_)	NFE (%_DM_)^7^	Crude lipid (%_DM_)	Energy (kJ/g DM)
5-days old larvae	26.9±0.3^bc^	13.1±0.0^c^	3.7±0.1^c^	55.4±0.0^b^	37.5±0.0^bc^	23.0±0.0^a^	8.5±0.0^e^	16.7±0.4^d^
**12-days old larvae, reared on**:								
CF^1^	29.5±0.2^a^	10.0±0.2^d^	5.1±0.1^a^	43.6±0.4^d^	31.9±0.3^d^	13.5±0.5^b^	32.9±0.7^a^	24.1±0.2^a^
MF^2^	26.2±0.6^abc^	9.0±0.3^d^	5.1±0.1^a^	43.4±1.2^d^	31.0±0.6^d^	20.8±1.0^a^	27.0±0.4^b^	23.2±0.5^a^
BG^3^	22.8±0.3^bc^	10.0±0.3^d^	4.7±0.2^ab^	59.8±0.4^a^	44.8±0.1^a^	7.7±0.7^cd^	22.4±0.2^cd^	21.6±0.2^b^
MM^4^	20.6±0.5^c^	22.9±0.7^a^	2.6±0.1^d^	48.4±1.4^c^	38.1±0.8^bc^	7.1±1.5^d^	21.6±0.6^d^	17.7±0.4^d^
RC^5^	29.1±0.3^a^	12.8±0.1^c^	4.7±0.3^ab^	51.5±0.3^bc^	40.5±1.1^b^	9.0±0.2^cd^	24.5±1.0^bc^	23.3±0.2^a^
SW^6^	21.8±2.5^bc^	15.8±0.5^b^	4.1±0.3^bc^	49.5±0.6^c^	36.2±0.9^c^	11.8±0.6^bc^	22.9±0.5^cd^	19.1±0.8^c^

^1^ Hornsyld Købmandsgaard A/S, Denmark;

^2^ Consisting of pea grits, wheat, chicken starter feed, sugar beet pellet, and vitamin-mineral mixture;

^3^ Carlsberg A/S, Denmark;

^4^ Danish Shellfish Centre, Denmark;

^5^ Emmelev A/S, Denmark;

^6^ Launis A/S, Denmark;

^7^ Nitrogen-free extract (%_DM_) = 100% − (crude lipid %_DM_ + crude protein %_DM_ + ash %_DM_).

### 3.3. Substrate composition

Table 2 shows the proximate composition of the rearing substrates before and after the seven days of rearing. The initial DM content ranged from 20.0 to 31.7% with marine-based substrates having the lowest DM content. Initial crude lipid was highest in rapeseed cake (12.7% DM) and lowest in mixed feed and mitigation mussels (3.4% DM). Shrimp waste had the highest initial crude protein content (37.6% DM) whilst being lowest in chicken feed, mixed feed and mitigation mussels (19.5–20.6% DM). Initial ash content showed a wide range within the substrates, ranging from 4.3–4.5% DM for brewer’s spent grain and mixed feed to 63.8% DM for mitigation mussels. Initial energy content varied from 11.4 kJ/g DM in mitigation mussels to 19.9 kJ/g DM in brewer’s spent grain.

**Table 2:** Proximate composition of rearing substrates at the start (initial, t = 0 days) and the end of the trial (final, t = 7 days). All values are shown as mean ± standard error (SE; n = 3) for the percentage of wet weight (%_WW_) or dry matter (%_DM_). Dissimilar lower case superscript letters represent significant differences between means within the same column (p < 0.05) whereas asterisks indicate a significant difference between initial and final substrate composition for the indicated parameter.

Rearing substrate	Dry matter (%_WW_)		Ash (%_DM_)		Crude protein (%_DM_)		NFE^7^ (%_DM_)		Crude lipid (%_DM_)		Calculated gross energy (kJ/g DM)^8^	
	Initial	Final	Initial	Final	Initial	Final	Initial	Final	Initial	Final	Initial	Final
CF^1^	31.2±0.1^a^	31.2±0.7^c^	5.4±0.1^c^*	7.8±0.2^d^*	19.5±0.1^d^	20.2±0.4^c^	70.6±0.2^a^	69.3±0.7^a^	4.5±0.1^d^*	2.6±0.0^c^*	17.1±0.1^c^	16.4±0.1^b^
MF^2^	28.7±0.1^b^*	30.8±0.5^c^*	4.5±0.2^c^*	5.9±0.4^d^*	20.6±0.0^d^*	25.1±1.2^b^*	71.5±0.2^a^*	65.9±1.8^a^*	3.4±0.0^e^	3.1±0.1^c^	17.5±0.0^c^	16.8±0.1^b^
BG^3^	31.2±0.1^a^*	39.8±1.5^b^*	4.3±0.0^c^*	6.3±0.1^d^*	26.1±0.3^c^	27.2±0.6^b^	59.8±0.5^b^	59.9±0.8^b^	9.8±0.3^b^*	6.6±0.5^a^*	19.9±0.1^a^*	18.8±0.2^a^*
MM^4^	26.2±0.7^c^*	47.9±1.5^a^*	63.8±1.5^a^*	77.7±1.2^a^*	19.9±0.3^d^*	8.0±0.1^d^*	16.8±1.6^e^*	9.8±1.5^d^*	3.4±0.0^e^*	0.9±0.1^d^*	11.4±0.1^e^*	3.7±0.9^d^*
RC^5^	31.7±0.2^a^*	39.2±0.5^b^*	7.0±0.0^c^*	13.7±0.1^c^*	30.9±0.1^b^	34.3±0.3^a^	49.4±0.1^c^*	44.7±0.2^c^*	12.7±0.0^a^*	7.2±0.1^a^*	19.4±0.1^b^*	17.0±0.1^b^*
SW^6^	20.0±0.2^d^	21.6±0.3^d^	31.2±0.4^b^*	53.5±0.1^b^*	37.6±0.4^a^*	28.0±0.1^b^*	23.5±0.5^d^*	13.9±0.6^d^*	7.4±0.1^c^*	5.3±0.0^b^*	12.1±0.1^d^*	9.9±0.1^c^*

^1^ CF = chicken feed;

^2^ MF = mixed feed;

^3^ BG = brewer’s spent grain;

^4^ MM = mitigation mussels;

^5^ RC = rapeseed cake;

^6^ SW = shrimp waste;

^7^ Nitrogen-free extract (NFE, %_DM_) = 100% − (crude lipid %_DM_ + crude protein %_DM_ + ash %_DM_);

^8^ Gross energy content was calculated using 23.66 MJ/kg protein, 39.57 MJ/kg lipid, and 17.17 MJ/kg nitrogen-free extract.

Generally, the proximate substrate composition expressed on DM changed during the trial period (Table 2). In all substrates, the ash content significantly increased over time and the DM content also increased in most substrates, except chicken feed and shrimp waste. Whereas the crude lipid, NFE, and gross energy contents were significantly lower in substrates at the end of the trial compared to the start, with a few exceptions. Crude protein content significantly decreased over time for the marine-based ingredients, whilst remaining relatively stable in the other substrates, except for mixed feed, which showed a significant increase.

Changes in substrate pH and temperature during the trial can be found in Table 3. Substrate temperature remained stable at approximately 28.0 °C throughout the trial for marine-based substrates whereas for mixed feed and chicken feed the temperature slowly increased to 36.9 and 39.9 °C, respectively, at the end of the trial. In comparison, temperatures peaked at 51.9 °C on day three for brewer’s spent grain and 46.5 °C on day five for rapeseed cake, and decreased to 37.3 and 36.7 °C, respectively, at the end of the trial. The pH for most substrates increased during the trial, except for chicken feed and shrimp waste. Chicken feed’s final substrate pH was 5.8, which was not significantly different from initial pH of 6.2, whereas for shrimp waste, the final pH of 7.4 was significantly lower than the initial pH of 8.4.

**Table 3:** Mean ± standard error (n = 3) temperature (°C) and pH of rearing substrates during the trial. Dissimilar lower case superscript letters represent significant differences between means within the same column and dissimilar capital letters represent significant differences between means within the same row of either temperature or pH.

Substrate	Trial length (days)							
	0	1	2	3	4	5	6	7
**Temperature (°C)**								
CF^1^	25.6±0.1^bcY^	28.3±0.0^bX^	27.9±0.1^cdX^	28.0±0.1^cdX^	31.7±0.9^bW^	30.5±0.1^bW^	36.4±0.6^bV^	39.9±0.3^aU^
MF^2^	26.5±0.2^bW^	27.6±0.4^bW^	27.7±0.3^dW^	26.2±0.6^dW^	30.6±0.5^bcV^	30.6±0.2^bV^	36.2±0.9^bU^	36.9±0.5^bU^
BG^3^	25.0±0.5^cZ^	37.0±1.1^aY^	41.5±0.3^bWX^	51.9±0.8^aU^	47.2±0.7^aV^	45.0±1.6^aVW^	38.2±0.6^bXY^	37.3±0.5^bY^
MM^4^	26.5±0.2^bX^	26.6±0.1^bWX^	25.4±0.2^eY^	27.5±0.1^cdUVW^	27.2±0.3^dVWX^	28.2±0.1^bU^	27.3±0.1^cUVWX^	27.6±0.2^cUV^
RC^5^	25.5±0.1^bcW^	37.1±0.2^aV^	44.7±0.9^aU^	43.9±1.2^bU^	45.2±0.2^aU^	46.5±0.2^aU^	45.2±1.6^aU^	36.7±0.9^bV^
SW^6^	30.3±0.3^aU^	28.7±0.2^bVWX^	30.0±0.5^cUVW^	30.2±0.6^cUV^	28.7±0.3^cdVWX^	27.5±0.2^bX^	27.9±0.2^cX^	28.6±0.2^cWX^
**pH**								
CF^1^	6.2±0.0^cU^	5.4±0.1^dV^	4.8±0.0^dW^	4.2±0.0^bX^	4.8±0.1^dW^	4.5±0.1^cWX^	5.5±0.3^cV^	5.8±0.1^dUV^
MF^2^	4.0±0.0^fW^	4.1±0.0^fW^	4.0±0.0^eW^	4.1±0.1^bW^	4.2±0.1^eVW^	4.5±0.0^cV^	5.0±0.2^cU^	5.0±0.1^eU^
BG^3^	4.8±0.0^eX^	5.1±0.0^eX^	5.9±0.1^cW^	7.0±0.2^aV^	8.6±0.1^aU^	8.6±0.1^aU^	8.8±0.0^aU^	9.0±0.1^aU^
MM^4^	6.9±0.1^bV^	6.3±0.0^bW^	6.7±0.1^bV^	6.7±0.1^aV^	6.9±0.1^cV^	7.3±0.1^bU^	7.3±0.0^bU^	7.6±0.0^cU^
RC^5^	5.8±0.0^dW^	5.6±0.0^cW^	6.3±0.2^bcW^	7.4±0.4^aV^	8.5±0.2^aU^	8.8±0.1^aU^	8.8±0.0^aU^	8.7±0.0^bU^
SW^6^	8.4±0.1^aU^	8.4±0.1^aU^	7.9±0.1^aV^	7.7±0.2^aVW^	7.5±0.0^bW^	7.3±0.0^bW^	7.3±0.0^bW^	7.4±0.0^cW^

^1^ CF = chicken feed;

^2^ MF = mixed feed;

^3^ BG = brewer’s spent grain;

^4^ MM = mitigation mussels;

^5^ RC = rapeseed cake;

^6^ SW = shrimp waste.

### 3.4. Amino acid profiles

The amino acid profile of initial and final larvae and their initial rearing substrates are shown in Table 4. The larval amino acid profile was significantly different between dietary treatments, although no significant correlations were detected between amino acids in the larvae and the rearing substrate. The most abundant amino acids in the larvae were glutamate + glutamine (3.4–6.2% DM), aspartate + asparagine (2.8–3.9% DM), and alanine (2.9–3.6% DM). Taurine and cysteine exhibited the lowest values of the detected amino acids in the larvae (0.0–1.1% DM and 0.1–0.2% DM, respectively) while cysteine and hydroxyproline contents were below detection levels in all larvae. The estimated nitrogen-to-protein conversion factor for the larvae ranged from 4.2 for initial larvae to 4.9 for final larvae reared on mitigation mussels and rapeseed cake.

**Table 4:** Amino acid composition of black soldier fly larvae at the start (5-days old larvae) and the end (12-days old larvae) of the trial after feeding different rearing substrates for 7 days and the amino acid composition of initial substrates. All values are shown as mean ± standard error (SE; n = 3) in percentage of dry matter (%_DM_), n.d. = not detected. Dissimilar lower case superscript letters represent significant differences between means within the same row for larvae data and capital superscript letters represent significant differences between means within the same row for initial substrate data (p < 0.05).

Identified amino acid (%_DM_)	5-days old larvae	12-days old larvae						Initial substrate					
		CF^4^	MF^5^	BG^6^	MM^7^	RC^8^	SW^9^	CF^4^	MF^5^	BG^6^	MM^7^	RC^8^	SW^9^
**Alanine**	3.3±0.0^b^	2.9±0.0^c^	3.2±0.1^bc^	3.8±0.1^a^	3.6±0.1^ab^	3.2±0.1^bc^	3.5±0.1^ab^	0.7±0.0^C^	0.8±0.0^C^	1.1±0.0^B^	0.9±0.0^C^	1.2±0.1^B^	1.7±0.0^A^
**Arginine**	2.3±0.0^ab^	1.7±0.0^de^	1.5±0.1^e^	2.4±0.0^a^	1.9±0.1^cd^	2.1±0.1^bc^	1.7±0.1^de^	1.1±0.0^C^	1.2±0.0^C^	1.2±0.0^C^	0.9±0.1^D^	1.6±0.1^B^	2.0±0.0^A^
**Asn + Asp** ^1^	3.7±0.0^b^	3.1±0.0^cd^	2.8±0.1^d^	4.1±0.1^a^	3.4±0.1^bc^	4.3±0.1^a^	3.4±0.1^bc^	1.6±0.0^C^	1.7±0.0^C^	1.6±0.0^C^	1.5±0.1^C^	2.1±0.1^B^	3.2±0.0^A^
**Cysteine**	0.2±0.0^a^	0.1±0.0^b^	0.1±0.0^b^	0.2±0.0^a^	0.1±0.0^ab^	0.1±0.0^ab^	0.1±0.0^b^	0.1±0.0^ABC^	0.1±0.0^BC^	0.2±0.0^AB^	0.1±0.0^C^	0.2±0.0^A^	0.2±0.0^AB^
**Gln + Glu** ^2^	6.2±0.0^a^	3.5±0.0^d^	3.4±0.0^d^	5.9±0.1^a^	4.9±0.1^bc^	4.6±0.1^c^	5.3±0.2^b^	3.5±0.0^C^	3.1±0.0^C^	5.4±0.1^A^	1.7±0.1^D^	4.3±0.2^B^	4.1±0.0^B^
**Glycine**	2.4±0.0^ab^	1.8±0.0^d^	1.8±0.0^d^	2.7±0.0^a^	2.2±0.1^bc^	2.3±0.1^bc^	2.1±0.1^c^	0.8±0.0^D^	0.8±0.0^D^	0.9±0.0^D^	2.1±0.1^A^	1.4±0.1^C^	1.7±0.0^B^
**Histidine**	1.8±0.0^c^	1.4±0.0^e^	1.4±0.0^e^	2.2±0.0^a^	1.5±0.0^de^	2.0±0.0^b^	1.6±0.0^d^	0.6±0.0^B^	0.6±0.0^B^	0.6±0.0^B^	0.4±0.0^C^	0.8±0.0^A^	0.8±0.0^A^
**Isoleucine**	1.8±0.0^abc^	1.5±0.0^d^	1.5±0.1^d^	2.0±0.0^a^	1.8±0.0^bc^	1.9±0.0^ab^	1.6±0.0^cd^	0.7±0.0^C^	0.7±0.0^C^	1.0±0.0^B^	0.6±0.0^C^	1.1±0.1^B^	1.3±0.0^A^
**Leucine**	3.0±0.0^ab^	2.4±0.0^d^	2.3±0.1^d^	3.2±0.0^a^	2.7±0.1^bc^	3.0±0.1^a^	2.5±0.1^cd^	1.3±0.0^B^	1.2±0.0^B^	1.8±0.0^A^	0.9±0.0^C^	1.9±0.1^A^	2.0±0.0^A^
**Lysine**	2.7±0.0^b^	2.3±0.0^c^	2.1±0.1^c^	3.1±0.1^a^	2.9±0.1^ab^	2.9±0.1^ab^	2.9±0.1^ab^	1.0±0.0^C^	1.0±0.0^C^	0.9±0.0^C^	0.8±0.0^C^	1.5±0.1^B^	1.9±0.0^A^
**Methionine**	0.8±0.0^a^	0.6±0.0^b^	0.5±0.0^b^	0.8±0.0^a^	0.7±0.0^a^	0.8±0.0^a^	0.7±0.0^a^	0.3±0.0^C^	0.2±0.0^D^	0.4±0.0^BC^	0.3±0.0^C^	0.4±0.0^B^	0.8±0.0^A^
**Phenylalanine**	1.5±0.0^b^	1.3±0.0^bc^	1.2±0.0^c^	1.8±0.0^a^	1.4±0.0^b^	1.9±0.1^a^	1.4±0.0^b^	0.8±0.0^C^	0.8±0.0^C^	1.4±0.0^A^	0.5±0.0^D^	1.1±0.1^B^	1.4±0.0^A^
**Proline**	2.6±0.0^ab^	2.3±0.1^cd^	2.3±0.0^cd^	2.7±0.0^a^	2.3±0.0^bc^	2.4±0.1^abc^	2.0±0.1^d^	1.1±0.0^C^	1.0±0.0^C^	2.6±0.1^A^	0.6±0.0^D^	1.7±0.1^B^	1.5±0.0^B^
**Serine**	2.0±0.0^ab^	1.5±0.0^d^	1.5±0.1^d^	2.1±0.0^a^	1.7±0.0^c^	1.8±0.1^bc^	1.7±0.0^c^	0.8±0.0^C^	0.8±0.0^C^	1.1±0.0^B^	0.8±0.0^C^	1.2±0.1^B^	1.6±0.0^A^
**Taurine**	n.d.	n.d.	n.d.	n.d.	1.1±0.0^a^	n.d.	0.1±0.0^b^	n.d.	n.d.	n.d.	0.8±0.0^A^	n.d.	0.1±0.0^B^
**Threonine**	1.9±0.0^ab^	1.5±0.0^de^	1.4±0.1^e^	2.0±0.0^a^	1.7±0.1^bcd^	1.8±0.0^abc^	1.6±0.0^cde^	0.7±0.0^C^	0.6±0.0^C^	0.9±0.0^B^	0.7±0.0^C^	1.3±0.1^A^	1.3±0.0^A^
**Tyrosine**	2.1±0.0^b^	2.1±0.0^b^	1.9±0.0^b^	2.8±0.1^a^	1.8±0.1^b^	2.9±0.1^a^	1.9±0.0^b^	0.6±0.0^D^	0.6±0.0^D^	0.9±0.0^BC^	0.8±0.0^CD^	1.0±0.1^B^	1.4±0.0^A^
**Valine**	2.4±0.0^bc^	2.0±0.0^de^	2.0±0.0^e^	2.9±0.0^a^	2.3±0.1^bcd^	2.6±0.0^b^	2.2±0.1^cde^	0.8±0.0^C^	0.8±0.0^C^	1.3±0.0^B^	0.7±0.0^C^	1.4±0.1^B^	1.6±0.0^A^
**Sum**	40.8±0.0^b^	31.9±0.3^d^	30.7±0.8^d^	44.8±0.1^a^	38.1±0.8^bc^	40.6±1.2^b^	36.2±0.9^c^	16.6±0.2^C^	15.9±0.1^C^	23.1±0.4^B^	15.0±0.6^C^	24.6±1.5^B^	28.6±0.2^A^
**Kp** ^3^	4.2±0.0^b^	4.6±0.0^ab^	4.5±0.2^ab^	4.7±0.0^ab^	4.9±0.1^a^	4.9±0.2^a^	4.6±0.1^ab^	-	-	-	-	-	-

^1^Asparagine + aspartic acid;

^2^ Glutamine + glutamic acid;

^3^ Nitrogen-to-protein factor (Kp) calculated using the formula described by Boulos et al. [28];

^4^ CF = chicken feed;

^5^ MF = mixed feed;

^6^ BG = brewer’s spent grain;

^7^ MM = mitigation mussels;

^8^ RC = rapeseed cake;

^9^ SW = shrimp waste.

### 3.5. Fatty acid profiles

Table 5 summarises the fatty acid profiles of the initial rearing substrates and harvested larvae. The most abundant fatty acids among the identified fatty acids in the larvae were 12:0 (5.3–37.6%), 16:0 (5.8–23.9%), 18:1n9 (12.0–46.8%), and 18:2n6 (1.6–29.9%). Larvae fed chicken feed, mixed feed, and brewer’s spent grain contained mostly saturated fatty acids (SFA, 47.5–60.8%) whereas larvae fed the other diets contained mostly mono-unsaturated fatty acids (MUFA, 41.3–53.7%). The polyunsaturated fatty acid (PUFA) content ranged from 14.0 to 34.3%. Hence, all larvae contained alpha-linolenic acid (ALA, 18:3n3) but generally in relatively minor amounts for most substrate treatments (< 1.0%) except for those reared on mitigation mussels (3.9%). Highly unsaturated fatty acids (HUFA) were abundant in larvae fed marine-based substrates whilst minimal in larvae fed the other substrates. Eicosapentaenoic acid (EPA, 20:5n3) and docosahexaenoic acid (DHA, 22:6n3) were found in larvae reared on the marine-based ingredients being mitigation mussels (5.3% and 0.9%, respectively) and shrimp waste (3.8% and 0.8%, respectively). A significant positive linear correlation was found between the fatty acid percentage in the rearing substrate and the larvae (n = 18) for C16:0 (r = 0.82, p < 0.001), C16:1n7 (r = 0.97, p < 0.001), C18:0 (r = 0.52, p = 0.026), C18:1n9 (r = 0.85, p < 0.001), C18:2n6 (r = 0.79, p < 0.001), C18:3n3 (r = 0.98, p < 0.001), C18:3n6 (r = 0.84, p < 0.001), C20:5n3 (r = 0.83, p < 0.001), C22:5n3 (r = 0.90, p < 0.001), and C22:6n3 (r = 0.95, p < 0.001).

**Table 5:** Fatty acid composition of black soldier fly larvae at the start (5-days old larvae) and the end (12-days old larvae) of the trial after feeding different rearing substrates for 7 days, as well as of the initial substrates. All values are expressed relative to the total identified fatty acids and shown as mean ± standard error (SE; n = 3), n.d. = not detected. Dissimilar lower case superscript letters represent significant differences between means within the same row for larvae data and capital superscript letters represent significant differences between means within the same row for initial substrate data (p < 0.05).

Identified fatty acid (%)	5-days old larvae	12-days old larvae						Initial substrate					
		CF^5^	MF^6^	BG^7^	MM^8^	RC^9^	SW^10^	CF^5^	MF^6^	BG^7^	MM^8^	RC^9^	SW^10^
**12:0**	5.3±0.2^g^	32.2±0.5^b^	37.6±0.5^a^	21.2±0.2^c^	7.5±0.3^f^	10.0±0.5^e^	16.9±0.5^d^	n.d.	n.d.	n.d.	n.d.	n.d.	n.d.
**14:0**	4.7±0.0^c^	6.6±0.2^b^	9.1±0.1^a^	4.5±0.0^c^	3.4±0.1^d^	2.0±0.1^e^	4.3±0.1^c^	0.5±0.0^BC^	0.2±0.0^CD^	0.4±0.0^BC^	1.0±0.2^B^	0.1±0.0^D^	1.4±0.1^A^
**16:0**	23.9±0.3^a^	9.1±0.2^d^	10.8±0.2^d^	17.0±0.3^b^	13.5±0.3^c^	5.8±0.5^e^	10.4±0.3^d^	23.3±0.1^B^	18.9±0.1^C^	27.0±0.1^A^	27.2±0.3^A^	5.6±0.0^E^	16.1±0.1^D^
**16:1 (n-7)**	3.7±0.1^d^	5.8±0.1^c^	5.3±0.3^c^	4.8±0.1^cd^	15.9±0.1^b^	2.5±0.1^e^	18.3±0.2^a^	0.3±0.0^D^	0.2±0.0^D^	0.3±0.0^D^	6.0±0.2^B^	0.6±0.0^C^	9.2±0.1^A^
**18:0**	6.1±0.1^a^	1.6±0.1^d^	2.2±0.1^cd^	2.6±0.0^bc^	2.9±0.1^b^	1.2±0.1^e^	1.8±0.1^d^	2.8±0.0^BC^	2.8±0.0^C^	1.6±0.0^D^	5.1±0.2^A^	1.0±0.1^E^	3.9±0.0^B^
**18:1 (n-9)**	26.4±0.2^b^	20.0±0.3^c^	15.7±0.4^d^	12.0±0.1^e^	20.7±0.3^c^	46.8±0.6^a^	26.9±0.5^b^	26.2±0.3^B^	22.5±0.2^C^	9.1±0.1^E^	8.6±0.4^E^	54.6±0.2^A^	15.8±0.0^D^
**18:2 (n-6)**	22.8±0.0^b^	20.1±0.5^bc^	13.6±0.6^d^	29.9±0.2^a^	11.3±0.3^d^	18.6±0.4^c^	1.6±0.1^e^	41.0±0.2^C^	45.0±0.2^B^	51.0±0.2^A^	5.3±0.1^E^	22.0±0.0^D^	1.7±0.0^F^
**18:3 (n-3)**	0.4±0.0^cd^	0.3±0.0^d^	0.4±0.0^cd^	0.5±0.0^bcd^	3.9±0.1^a^	0.8±0.0^b^	0.7±0.0^bc^	<0.1^D^	<0.1^D^	<0.1^D^	3.0±0.1^A^	0.1±0.0^C^	0.7±0.1^B^
**18:3 (n-6)**	2.2±0.0^cd^	1.8±0.0^d^	2.5±0.1^bc^	3.0±0.0^b^	2.7±0.1^b^	6.2±0.1^a^	0.5±0.0^e^	3.0±0.0^D^	6.7±0.1^B^	6.0±0.1^C^	3.2±0.0^D^	8.1±0.0^A^	0.8±0.1^E^
**20:5 (n-3)**	<0.1^c^	<0.1^c^	<0.1^c^	<0.1^c^	5.3±0.0^a^	<0.1^c^	3.8±0.1^b^	n.d.	n.d.	0.3±0.0^C^	7.4±0.2^B^	0.1±0.0^D^	15.2±0.1^A^
**22:5 (n-3)**	n.d.	n.d.	n.d.	n.d.	0.8±0.0	n.d.	0.8±0.0	n.d.	n.d.	n.d.	0.6±0.0^B^	n.d.	1.3±0.0^A^
**22:6 (n-3)**	n.d.	n.d.	n.d.	n.d.	0.9±0.0	n.d.	0.8±0.0	n.d.	n.d.	n.d.	8.8±0.2^B^	n.d.	11.8±0.1^A^
**∑SFA** ^1^	41.7±0.2^c^	50.4±1.1^b^	60.8±0.8^a^	47.5±0.4^bc^	28.7±0.6^d^	19.9±1.0^e^	34.3±1.0^d^	27.8±0.3^B^	22.9±0.1^C^	30.3±0.2^B^	36.0±0.8^A^	7.7±0.2^D^	23.2±0.2^C^
**∑MUFA** ^2^	32.2±0.1^c^	27.0±0.4^d^	22.3±0.7^e^	18.3±0.2^f^	41.3±0.3^b^	53.7±0.6^a^	51.7±0.8^a^	28.1±0.1^C^	25.3±0.2^D^	11.8±0.1^E^	29.7±0.6^C^	61.7±0.2^A^	39.4±0.2^B^
**∑PUFA** ^3^	25.8±0.1^b^	22.5±0.7^bc^	16.8±0.8^d^	34.3±0.2^a^	20.1±0.4^cd^	26.6±0.6^b^	5.8±0.5^e^	44.0±0.2^C^	51.7±0.3^B^	57.3±0.3^A^	14.4±0.7^E^	30.5±0.1^D^	5.4±0.4^F^
**∑HUFA** ^4^	0.1±0.0^c^	<0.1^c^	<0.1^c^	0.1±0.0^c^	9.8±0.2^a^	<0.1^c^	8.2±0.3^b^	<0.1^DE^	<0.1^E^	0.6±0.1^C^	18.9±0.5^B^	0.1±0.0^D^	32.0±0.2^A^
**∑n-3**	0.5±0.0^c^	0.4±0.0^c^	0.5±0.0^c^	0.5±0.0^c^	13.2±0.2^a^	1.0±0.0^c^	6.7±0.3^b^	<0.1^E^	<0.1^E^	0.6±0.0^C^	22.3±0.4^B^	0.4±0.0^D^	29.7±0.1^A^
**∑n-6**	25.0±0.1^b^	21.9±0.7^b^	16.1±0.8^c^	33.0±0.2^a^	14.9±1.8^c^	24.8±0.4^b^	5.1±0.2^d^	44.0±0.2^C^	51.7±0.3^B^	57.3±0.2^A^	10.7±0.1^E^	30.2±0.1^D^	6.2±0.1^F^

^1^ ∑SFA = sum of all identified saturated fatty acids;

^2^ ∑MUFA = sum of all identified mono-unsaturated fatty acids;

^3^ ∑PUFA = sum of all identified poly-unsaturated fatty acids (including n-3 and n-6);

^4^ ∑HUFA = sum of all identified highly unsaturated fatty acids (≥20:0 with 3 or more double bonds, including n-3 and n-6);

^5^ CF = chicken feed;

^6^ MF = mixed feed;

^7^ BG = brewer’s spent grain;

^8^ MM = mitigation mussels;

^9^ RC = rapeseed cake;

^10^ SW = shrimp waste.

## 4. Discussion

This study was designed to investigate whether BSF larval macronutrient composition could be modified and potentially tailored for food and feed purposes via the rearing substrate. The selected rearing substrates included both biowaste and by-products locally available in Denmark. It was observed that the percentages of all investigated larval composition parameters (protein, lipid, amino acids, fatty acids, ash, and chitin) on DM basis were significantly different between dietary treatments. In particular, larvae reared on marine-based ingredients had a relatively higher share of omega-3 fatty acids, especially EPA, than other treatment groups, reflecting the presence of these fatty acids in the substrates. The findings are consistent with previous studies using other marine-based substrates such as algae [33], fishery waste [15, 34] and salmon oil [35]. Similar to the current study, EPA was the main omega-3 fatty acid accumulating in larvae reared on fishery waste, followed by ALA and subsequently DHA [34]. The accumulation of omega-3 fatty acids in the larvae can increase their value and widen their applications, whilst valorising waste streams. The finding that MUFA was the main fatty acid class in larvae reared on mitigation mussels, shrimp waste, and rapeseed cake whilst SFA was the most abundant class for larvae fed the other substrates contradicts previous work showing that SFA was the main fatty acid class in harvested BSF larvae independent of the tested substrate [14, 16, 36]. However, it accords with the findings by Starcevic et al. [37]. A possible explanation for these contradictory findings could relate to differences in the nutritional composition of the substrate and particularly the digestible carbohydrate content [38]. Black soldier fly larvae can produce SFA from digestible carbohydrates *de novo* and/or bioaccumulate SFA from the rearing substrate, depending on the fatty acid [39]. The most abundant fatty acid in the larvae reared on the control substrates and brewer’s spent grain was lauric acid (C12:0), which has been previously shown to be produced exclusively *de novo* by the larvae, using carbohydrates as a source of acetyl-CoA [39]. The lower NFE content in rapeseed cake and marine-based substrates, when compared to the other substrates, could have led to a deprived availability of acetyl-CoA and reduced the *de novo* production of SFA such as lauric acid.

Compared to fatty acids, total larval protein and lipid content at harvest did not reflect the nutritional composition of the substrate. There were, however, significant differences in the proximate composition of harvested larvae between dietary treatments likely reflecting dissimilar development stages. Previous work has found that the length of the larval stage depends on the nutrient availability and the time needed to obtain sufficient nutrients required for the next life stages [20]. When nutrients are limited, larvae can prolong their larval stage by up to 70 days [40]. In the current trial, all larvae were harvested after 12 days, independent of the rearing substrate, and larvae reared on biowaste and by-products may have been in earlier instars when compared to the control treatments. This hypothesis is supported by the visual observations that larvae reared on the control substrates were darker in colour, indicating that they were closer to pupation [41]. Furthermore, the chitin content is typically higher in later larval instars [42], and larvae fed the control diets had a higher chitin content corroborating that they were closer to pupation than the other treatment groups. Lastly, larvae closer to pupation typically contain less crude protein [43], which was also observed for larvae fed the control diets. In general, therefore, it seems that the larval composition of protein, lipid and chitin are affected by the rearing substrate but mostly due to differences in larval development, as reflected in the growth rate, rather than directly reflecting the substrate composition.

The presence of chitin and other nitrogen-containing compounds (e.g. nucleic acids, uric acid, urea, and ammonia) may result in an overestimation of the crude protein content in BSF when using the standard nitrogen-to-protein conversion factor Kp of 6.25. A more precise protein content may therefore be obtained by determining the total amino acid content or by using an estimated Kp factor for BSF of 4.67 as suggested by Janssen et al. [44]. In comparison, in the current study, Kp values ranged from 4.24 to 4.92, depending on the type of larvae sample. It is expected that the true Kp values are slightly higher as tryptophan was not analysed. Tryptophan is, however, one of the least abundant amino acids in BSF larvae [8] and the effect on the Kp estimations is presumably minimal. The average Kp value in the current study was 4.62 ± 0.09, which is in line with that of Janssen et al. [44], confirming that a Kp value of 6.25 largely overestimates the crude protein content in BSF. Interestingly, the calculated NFE fraction, using a Kp factor of 6.25, in BSF larvae (ranging from 7.1–23.0% DM) was much higher than the chitin fraction. These results indicate that part of the NFE value includes other non-identified organic compounds that could be, amongst others, phenols and nucleic acids, as suggested by Janssen et al. [44].

With regards to amino acids, larval profiles were slightly affected by the rearing substrates but mostly seemed tightly regulated within narrow ranges, as previously reported by Oonincx and Finke [45], who concluded that the amino acid composition is mostly unaffected by diet or life stages. It was therefore interesting that taurine was detected in larvae fed marine-based ingredients but not in larvae fed the other substrates. Taurine concentrations are reportedly very low in BSF larvae and prepupae [42], and the detection in larvae fed marine-based ingredients may well have been a result of substrate remnants in their intestinal tract (gut load) rather than *de novo* synthesis as the larvae were not starved before harvesting. Taurine is an essential amino acid in e.g. cats [46], a semi-essential amino acid in e.g. fish [47], and a feed attractant in e.g. lobsters [48], and it would therefore be interesting to further research the role of gut load on the possibility of increasing larval taurine content. Similar to taurine, the higher ash content in larvae fed mitigation mussels and shrimp waste when compared to other substrates was probably also a reflection of gut load due to the presence of crushed shell remnants in the larvae’s intestinal tract.

Except for rapeseed cake, growth performance was generally poor for larvae reared on single biowaste or by-product substrates compared to the control diets. This was not surprising given that the single substrates were not optimised to maximise larval performance. It was therefore unexpected that larvae performed relatively well on rapeseed cake, also considering the high substrate temperatures measured (max. 46.5 °C) (Table 3), whilst ambient temperature was kept at the optimal BSF larvae rearing temperature of 27 °C [49]. High substrate temperatures (up to 45 °C) have been also measured during biodegradation of corn bran, soybean bran and corn bran, or soybean bran and corn hull by housefly (*Musca domestica*) and BSF [50]. The large difference in ambient and substrate temperature could be due to biological activity of larvae and likely microbes [50, 51], as substrates were not autoclaved before the start of the trial. Hence, the steep temperature increase during the first days of the trial could indicate the degradation of simple compounds (e.g. sugars, amino acids, and protein) by bacteria and fungi, which has been suggested to promote larval growth [50]. Additionally, as BSF are ectothermic, they largely depend on ambient temperatures to regulate their metabolism [52], and high substrate temperatures can support larval nutrient digestion, as BSF have several digestive enzymes with an optimal temperature of ~47 °C [53]. However, even higher temperatures can reduce digestive enzyme activity and have negative implications for larval performance and survival, as might be the case with larvae reared on brewer’s spent grain, where extremely high substrate temperatures were measured (max. 51.9 °C). Although the lethal upper substrate temperature limit for BSF larvae is unknown, the lethal upper environmental temperature threshold reportedly ranges from 37.2–44.0 °C at 70% relative humidity [54]. The much higher substrate temperature may have resulted from the high fibre content in brewer’s spent grain acting as an insulator for metabolic heat production of larvae and possibly microbes, as previously described [51]. Secondly, the high lignin content in brewer’s spent grain (10–27% DM) might have impaired larval growth as lignin is assumed to be poorly digestible by BSF larvae [55–57]. A previous study found that lignin digestion by BSF larvae fed a mixture of rice straw and restaurant waste was close to zero, whilst after the addition of a microbe and enzyme mixture, lignin digestibility increased to 8.8% [57]. This finding indicates that pre-treatment of substrates containing lignin with microorganisms and/or enzymes could aid in the conversion of complex carbohydrates into more easily digestible nutrients for the larvae.

A potential explanation for the poor growth of larvae fed exclusively marine-based substrates could be their limited content of digestible carbohydrates, the main energy source for most insect species [58]. The lack of digestible carbohydrates presumably forced larvae to use crude protein as their main energy source as deduced from the decline in substrate crude protein content during the trial, compared to an increase in the other substrates. The use of protein as an energy source is more energy costly than that of carbohydrates [59], which could explain their poorer performance. Additionally, the estimated gross energy content in the marine-based substrates was significantly lower when compared to the other substrates. As energy is needed for growth, part of the reduced growth could also be due to the limited dietary energy density of the marine-based substrates.

Serving as a benchmark substrate for optimal BSF larval performance and for comparison to other studies, a commercial chicken feed was included as a control diet as BSF larvae typically have a higher performance on chicken feed than on other substrates [8]. However, using a high-quality rearing substrate for producing black soldier fly at an industrial scale is not sustainable from an environmental and economical point of view, and contradicts concepts of waste valorisation and circular economy [60]. The current study therefore, investigated the use of the mixed feed substrate, aiming to achieve similar performance as chicken feed, but partly replaced using by-products. However, despite a similar macronutrient composition and inclusion of vitamins and mineral mixture, the mixed feed resulted in a lower final body weight of the larvae, suggesting a lack or mismatch of essential nutrients. These findings emphasise that the nutritional requirements of BSF larvae are currently unknown and need to be identified to be able to optimally use waste streams to achieve maximal larvae yield with a minimal environmental footprint.

## 5. Conclusions and future work

The current study found that BSF larvae were able to grow on a wide range of biowaste products and by-products. However, the rearing substrate largely affected larval body composition. The fatty acid composition of the larvae could be greatly tailored by the rearing substrate with larvae accumulating, amongst others, omega-3 fatty acids and oleic acid, which can improve the nutritional and economical value of the larvae and widen their applications, for example in aquaculture. Additionally, larval ash content was positively correlated to the ash content in the rearing substrate possibly due to an accumulation of ash in the larval intestinal tract. It might therefore be necessary to modify the substrate ash content depending on further food and feed applications. For the other investigated parameters (protein, amino acid, chitin, lipid), differences between dietary treatments were rather related to differences in larval development. Larvae fed the different rearing substrates were likely in different instars at the time of harvest and therefore showed differences in these parameters, and when wanting to tailor those, it might be more appropriate to adjust the larval harvest time than the substrate composition. More work is needed to determine larvae’s nutritional requirements for macro and micronutrients, applications of rearing substrate pre-treatments using microorganisms and/or enzymes, and optimal physical substrate properties to efficiently use waste streams to produce BSF larvae.

## Supporting information

S1 Fig Larval protein content over time. Mean ± standard error (n = 3) larval protein content (% dry matter, DM) of black soldier fly larvae over time reared on six different rearing substrates: chicken feed, mixed feed, brewer’s spent grain, mitigation mussels, rapeseed cake, and shrimp waste.
(TIF)

S2 Fig Larval lipid content over time. Mean ± standard error (n = 3) larval lipid content (% dry matter, DM) of black soldier fly larvae over time reared on six different rearing substrates: chicken feed, mixed feed, brewer’s spent grain, mitigation mussels, rapeseed cake, and shrimp waste.
(TIF)
