# Supplementary figures and images for: Biowaste and by-products as rearing substrates for black soldier fly (Hermetia illucens) larvae: Effects on larval body composition and performance
Source: PLoS One. 2022 Sep 29;17(9):e0275213. doi: 10.1371/journal.pone.0275213 (PMC9521838; doi:10.1371/journal.pone.0275213)

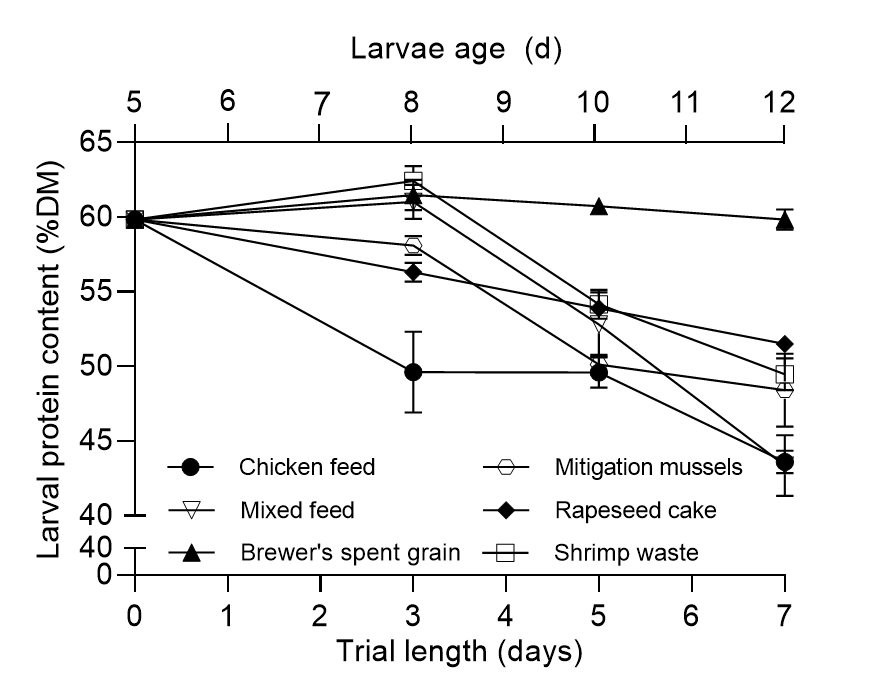

Supplement: S1 Fig — Mean ± standard error (n = 3) larval protein content (% dry matter, DM) of black soldier fly larvae over time reared on six different rearing substrates: chicken feed, mixed feed, brewer’s spent grain, mitigation mussels, rapeseed cake, and shrimp waste. (TIF) [file pone.0275213.s001.tif]

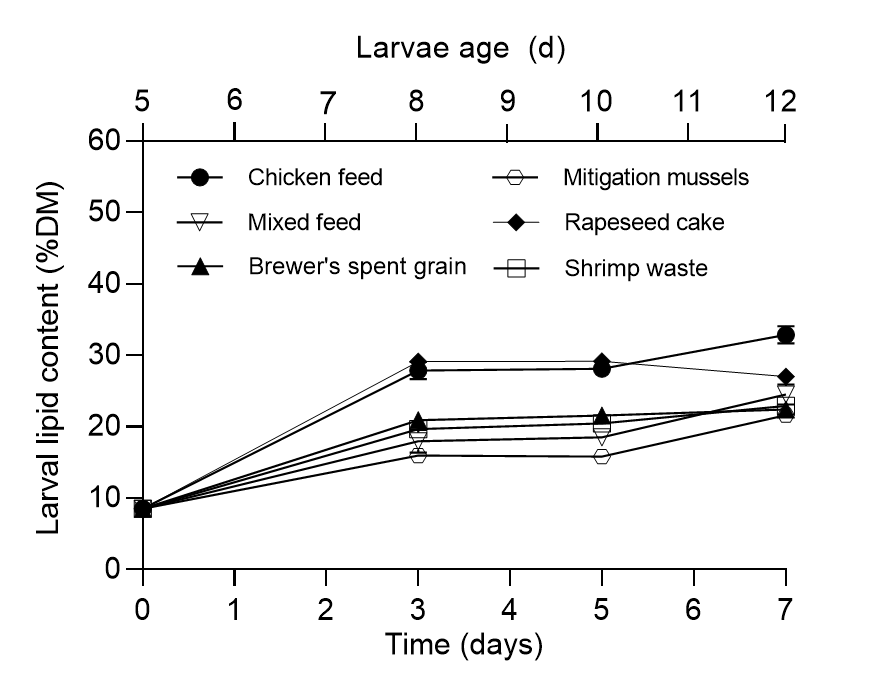

Supplement: S2 Fig — Mean ± standard error (n = 3) larval lipid content (% dry matter, DM) of black soldier fly larvae over time reared on six different rearing substrates: chicken feed, mixed feed, brewer’s spent grain, mitigation mussels, rapeseed cake, and shrimp waste. (TIF) [file pone.0275213.s002.tif]
